# Genomic islands from five strains of *Burkholderia pseudomallei*

**DOI:** 10.1186/1471-2164-9-566

**Published:** 2008-11-27

**Authors:** Apichai Tuanyok, Benjamin R Leadem, Raymond K Auerbach, Stephen M Beckstrom-Sternberg, James S Beckstrom-Sternberg, Mark Mayo, Vanaporn Wuthiekanun, Thomas S Brettin, William C Nierman, Sharon J Peacock, Bart J Currie, David M Wagner, Paul Keim

**Affiliations:** 1 Department of Biological Sciences, Northern Arizona University, Flagstaff, AZ, 86011-5640, USA; 2 The Translational Genomics Research Institute, 445 N. Fifth St. Phoenix, AZ, 85004, USA; 3 Menzies School of Health Research, Charles Darwin University, Northern Territory, Australia; 4 Mahidol-Oxford Research Unit, Mahidol University, Bangkok, Thailand; 5 Los Alamos National Laboratory, Los Alamos, NM, 87545, USA; 6 J. Craig Venter Institute, 9704 Medical Center Drive, Rockville, MD 20850, USA; 7 George Washington University, 2300 Eye Street, NW, Washington DC, 20037, USA

## Abstract

**Background:**

*Burkholderia pseudomallei *is the etiologic agent of melioidosis, a significant cause of morbidity and mortality where this infection is endemic. Genomic differences among strains of *B. pseudomallei *are predicted to be one of the major causes of the diverse clinical manifestations observed among patients with melioidosis. The purpose of this study was to examine the role of genomic islands (GIs) as sources of genomic diversity in this species.

**Results:**

We found that genomic islands (GIs) vary greatly among *B. pseudomallei *strains. We identified 71 distinct GIs from the genome sequences of five reference strains of *B. pseudomallei*: K96243, 1710b, 1106a, MSHR668, and MSHR305. The genomic positions of these GIs are not random, as many of them are associated with tRNA gene loci. In particular, the 3' end sequences of tRNA genes are predicted to be involved in the integration of GIs. We propose the term "tRNA-mediated site-specific recombination" (tRNA-SSR) for this mechanism. In addition, we provide a GI nomenclature that is based upon integration hotspots identified here or previously described.

**Conclusion:**

Our data suggest that acquisition of GIs is one of the major sources of genomic diversity within *B. pseudomallei *and the molecular mechanisms that facilitate horizontally-acquired GIs are common across multiple strains of *B. pseudomallei*. The differential presence of the 71 GIs across multiple strains demonstrates the importance of these mobile elements for shaping the genetic composition of individual strains and populations within this bacterial species.

## Background

*Burkholderia pseudomallei *is the causative agent of melioidosis, an important tropical disease affecting people in Southeast Asia and the tropical "Top End" of northern Australia. Within these regions, this Gram-negative bacterium exists in the environment as a soil saprophyte and accounts for 20% of community-acquired septicemias and may cause death in 40% of treated patients [1]. Re-infection and relapse are common in melioidosis patients [2,3]. Infection can occur when contaminated soil or water comes into contact with breaks in the skin; this is a common infection route for rice farmers in northeastern Thailand [4,5]. Inhalation of contaminated dust has also been confirmed as a route of infection, including U.S. helicopter pilots during the Vietnam War [6]. Because of the high inhalational risk, the U.S. Centers for Disease Control and Prevention have classified *B. pseudomallei *as a Category B Select Agent [7]. Various clinical manifestations are associated with melioidosis, ranging from subclinical involvement to symptomatic characteristics that may include localized cutaneous infection, acute pulmonary infection, bacteremia, and disseminated infection [1,8,9]. Cheng and Currie [10] suggested that this variation in clinical presentation may be caused by one or more of three factors: variation in mode of acquisition, variation in host immune response, or variation among bacterial strains, including presence/absence of virulence factors.

Many challenges exist for the control and prevention of melioidosis [9]. At present, cellular and molecular mechanisms associated with these diverse clinical manifestations are not fully understood. Vaccine development and better therapeutics are necessary to prevent and treat melioidosis. However, knowledge of the relationship between hosts and pathogen is still limited. This has hindered improved vaccine and therapeutic developments, which require a full understanding of genomics and bacterial pathogenesis.

After the first genome of *B. pseudomallei *K96243 was released in 2004 [11], subsequent studies capitalized upon this foundation, which lead to enhanced genetic and genomic analyses that have facilitated a better understanding of this organism. As additional genomic sequences have been generated, striking differences have been observed. For example, two mutually exclusive gene cassettes, termed "BTFC and YLF", have been described that are dissimilar in their geographical distribution [12].

It is well established that *B. pseudomallei *contains an "open" genome [13] that recombines at a high frequency, leading to great intra-species diversity within and among pathogen populations. In recent unpublished analyses, we have found that when compared to existing genomic sequences, new *B. pseudomallei *genome sequences can contain as much as 500 Kb of additional genomic material in the form of blocks of novel DNA known as genomic islands. Thus, we hypothesize that the primary differences among *B. pseudomallei *genomes are horizontal gene transfer events from diverse bacterial or phage origins. Horizontal gene transfer involves the incorporation of genetic elements, perhaps directly into the genome where they form genomic islands [14]. Currently, very little is known about fitness in *B. pseudomallei*. It seems likely that the genes contained in genomic islands may generate unique phenotypes and affect bacterial fitness, such as the interaction of bacterial cells with their surrounding environment. Fitness phenotypes could range from the ability to survive under extreme environmental conditions to the ability to defeat host immune system defenses.

Here, we describe diversity among *B. pseudomallei *genomes in terms of identification and differential possession of genomic islands. Nomenclature for genomic islands is proposed, and the specific mechanism behind the genetic recombination is explained in detail. Finally, we present the frequency distributions of a focused group of interesting genomic islands across a large, diverse collection of *B*. *pseudomallei *isolates.

## Results and discussions

### Identification of genomic islands in *B. pseudomallei*

#### i) Genomic comparison of five *B. pseudomallei *strains

We identified genomic islands in *B. pseudomallei *from a set of 5 diverse reference genomes: strains K96243, 1710b, 1106a, MSHR668, and MSHR305 (Table 1). All 5 strains were isolated from melioidosis patients in Thailand or Australia. Clinical manifestations and severity of disease caused by these 5 strains were varied. Thai patients infected by strains K96243, 1710b and 1106a exhibited classical melioidosis manifestations, including lung and liver abscesses, and septicemia. In contrast, Australian patients infected by strains MSHR668 and MSHR305 had the relatively rare melioidosis encephalomyelitis and neurological involvement. Patients infected with strains K96243, 1710b, and MSHR305 died. Detailed clinical information associated with all five isolates, as well as their genomic features, is summarized in Table 1.

**Table 1:** Information about the five *B. pseudomallei *strains utilized in the genomic island analyses.

**Strain**	**Clinical Information**	**Genomic information**		**Source**
			
		**Size**	**Accession no. & Genome Center**	
**K96243**	Isolated in 1996 in Khon Kaen, Thailand from a 34 year old female diabetic patient with a clinical history of short incubation, septicemic infection, and rapid progression to death.	Chr1: 4.07 Mb; Chr2: 3.17 Mb	BX571965 and BX571966 Sanger Institute	Dr. S. Songsivilai*
**1710b**	Isolated in 1999 in Ubon Ratchathani, Thailand from blood collected from a 55 year old male patient with known primary melioidosis in 1996. This second episode represented relapse with the same strain. The patient died.	Chr1: 4.13 Mb; Chr2: 3.18 Mb	CP000124 and CP000125 JCVI	Dr. S. Peacock
**1106a**	Isolated in 1993 in Ubon Ratchathani, Thailand from pus aspirated from liver abscess from a 23-year old female rice farmer. The patient survived.	Chr1: 3.99 Mb; Chr2: 3.10 Mb	CP000572 and CP000573 JCVI	Dr. S. Peacock
**MSHR668**	Isolated in 1995 in Darwin, Australia from the blood of a 53 year old male patient with severe melioidosis encephalomyelitis. The patient survived.	Chr1: 3.91 Mb; Chr2: 3.13 Mb	CP000570 and CP000571 JCVI	Dr. B. Currie
**MSHR305**	Isolated in 1994 from an autopsy sample from the brain of a fatal melioidosis encephalomyelitis case at the Royal Darwin Hospital, Northern Territory, Australia. Neurological melioidosis is rare, occurring only in approximately 5% of diagnosed cases.	36 contigs 7,453,647 nt	AAYX00000000 JGI	Dr. B. Currie

*Described in [11].

JCVI (J. Craig Venter Institute); JGI (Joint Genome Institute)

The five genomes were compared using Artemis Comparison Tool (ACT) [15], which enabled genomic alignment and visualization of BLASTN results. We confirmed that all genome sequences contained 2 chromosomes, except the genome sequence of MSHR305, which was incomplete at the time of writing this manuscript. To facilitate genomic comparison with the other 4 strains, we created 2 artificial chromosomes from the genome contigs of strain MSHR305 [see Additional file 1: Figure S1a and S1b for circular genomic structures], recognizing that this genomic arrangement is not confirmed.

In general, we identified genomic islands by identifying variable regions among the five genomes and determining if any of these regions met the general criteria previously described by Holden *et al*. in their analysis of strain K96243 [11], or the criteria described by Hacker and Kaper [16]. These criteria included: size of large inserts, distinct %G+C compared to the rest of the genome, and the presence of mobility genes. It is important to note that across the 5 strains multiple GIs were often present at the same genomic location (Table 2). In practice, our identification of genomic islands involved four main steps.

**Table 2:** 71 GIs identified among five strains of *B. pseudomallei*.

**Strains**	K96243*	**1710b**	**1106a**	**MSHR668**	**MSHR305**	**Strains with GIs**	**Unique GIs**	**% Unique**
**Chromosome 1**	**GI1**	**GI1**	**GI1**	GI1.1	-	4	2	50%
	-	-	-	GI1a	-	1	1	100%
	GI2	-	-	-	-	1	1	100%
	GI3	GI3.1	-	-	-	2	2	100%
	-	-	-	-	GI3a	1	1	100%
	GI4	GI4.1	GI4.2	GI4.3	GI4.4	5	5	100%
	GI5	-	-	-	-	1	1	100%
	-	GI5a	**GI5a.1**	**GI5a.1**	GI5a.2	4	3	75%
	**GI6**	-	-	-	**GI6**	2	1	50%
	-	-	GI6a	-	-	1	1	100%
	-	GI6b	-	-	-	1	1	100%
	GI7	GI7.1	GI7.2	GI7.3	GI7.4	5	5	100%
	GI8	GI8.1	GI8.2	-	GI8.3	4	4	100%
	-	-	-	GI8a	-	1	1	100%
	-	**GI8b**	**GI8b**	-	-	2	1	50%
	-	-	-	-	GI8c	1	1	100%
	-	-	-	-	GI8d	1	1	100%
	GI9	-	-	-	-	1	1	100%
	-	-	-	GI9a	-	1	1	100%
	-	-	-	GI9b	-	1	1	100%
	-	-	-	GI9c	GI9c.1	2	2	100%
	**GI10**	GI10.1	GI10.2	**GI10**	GI10.3	5	4	80%
	GI11	-	GI11.1	-	GI11.2	3	3	100%
	GI12	GI12.1	GI12.2	GI12.3	GI12.4	5	5	100%
	-	-	GI12a	-	-	1	1	100%

**Chromosome 2**	GI13	GI13.1	GI13.2	GI13.3	GI13.4	5	5	100%
	**GI14**	**GI14**	**GI14**	GI14.1	**GI14**	5	2	40%
	-	-	-	-	GI14a	1	1	100%
	GI15	-	-	-	GI15.1	2	2	100%
	-	GI15a	-	-	-	1	1	100%
	-	-	-	-	GI15b	1	1	100%
	-	-	-	GI15c	-	1	1	100%
	-	-	-	-	GI15d	1	1	100%
	-	**GI15e**	**GI15e**	**GI15e**	**GI15e**	4	1	25%
	GI16	**GI16.1**	**GI16.1**	-	GI16.2	4	3	75%
	-	-	-	GI16a	-	1	1	100%
	**GI16b**	**GI16b.1**	**GI16b.1**	**GI16b**	**GI16b.1**	5	2	40%

**Total GIs**	17	16	16	17	21			
**Unique GIs**	12	10	9	13	17			
**% Unique**	71%	63%	56%	76%	81%			

**Note: **Rows represent 37 different genomic locations where GIs were found in at least one strain. These positions are listed according to the relative location in strain K96243. Different GI names indicate different gene content [see Additional file 3]. GIs shared among strains at the same relative genomic location are indicated (**bold**). * GIs1-16 in strain K96243 were previously described [11], whereas GI16b is reported here for the first time.

First, we determined if the 16 specific GIs previously identified in K96243 [11] were present in the four other strains. In K96243, 12 of these GIs are found on chromosome 1 and four are found on chromosome 2. Using the genomic locations of these GIs in K96243 as a reference, we performed pair-wise comparisons between K96243 and the other four strains and noted the differential presence of the 16 GIs in the other four strains. These 16 specific GIs were uncommon in the other 4 *B. pseudomallei *genomes: only four of these GIs (GI1, GI6, GI10, and GI14) were found in at least one of the other four strains, and none were found in all of the other strains (Table 2).

Second, we identified new, distinct GIs that were present at the same 16 genomic locations where GIs were previously found in K96243. To accomplish this, we used ACT to align the genomes of all five strains in the following order: K96243 vs. 1710b vs. 1106a vs. MSHR668 vs. MSHR305. In addition to identifying structural variation among any GIs present at these 16 locations, this alignment also allowed us to locate integration sites. Even when there is more than one GI found at the same relative genomic location, the integration sites were all similar across the five strains and these integrations sites were often associated with tRNA genes (Table 3). The genomic locations of GI2, GI5, and GI9 in K96243 did not contain GIs in any of the other four strains. One of the other locations, corresponding to GI6 in K96243, contained an identical GI in just strain MSHR305. However, the GIs located at the 13 other locations were quite diverse: at four of these locations each of the five strains possessed a distinct GI containing a different set of genes (Table 2).

**Table 3:** GIs located next to tRNA genes in five *B. pseudomallei *strains.

	**GIs**	**GIs located next to tRNA genes**					**tRNA-SSR**
			
		**K96243**	**1710b**	**1106a**	**MSHR668**	**MSHR305**	
**Chromosome 1**	GI1a				X		
	GI2	X					tRNA-Phe
	**GI4**	X					**tRNA-Met**
	**GI4.1**		X				**tRNA-Met**
	**GI4.2**			X			**tRNA-Met**
	GI4.3				X		
	**GI4.4**					X	**tRNA-Met**
	GI5	X					tRNA-Leu
	GI5a		X				
	GI5a.1			X	X		
	GI5a.2					X	
	GI6b		X				tRNA-Pro
	GI7	X					
	**GI7.1**		X				**tRNA-Arg**
	GI7.2			X			
	**GI7.3**				X		**tRNA-Arg**
	GI7.4					X	
	GI8d					X	tRNA-Cys
	GI9	X					tRNA-Ser
	GI9a				X		
	GI9b				X		tRNA-Gly
	**GI9c**				X		**tRNA-Pro**
	**GI9c.1**					X	**tRNA-Pro**
	**GI10**	X			X		**tRNA-Thr**
	GI10.1		X				
	**GI10.2**			X			**tRNA-Thr**
	**GI10.3**					X	**tRNA-Thr**
	**GI11**	X					**tRNA-Ala**
	GI11.1			X			
	**GI11.2**					X	**tRNA-Ala**
	**GI12**	X					**tRNA-Arg**
	GI12.1		X				
	GI12.2			X			
	**GI12.3**				X		**tRNA-Arg**
	GI12.4					X	

**Chromosome 2**	GI13	X					tRNA-Ser
	GI13.1		X				
	GI13.2			X			
	GI13.3				X		
	GI13.4					X	
	GI15d					X	

**Note: **The specific tRNA genes that facilitate tRNA-site specific recombination (tRNA-SSR) at some of these genomic locations are listed. In many cases tRNA-SSR (**bold**) has facilitated the insertion of multiple, distinct GIs in different strains at the same genomic location.

Third, we identified new, distinct GIs that were present at genomic locations other than the 16 previously described from K96243 [11]. These new GIs were identified by searching for integrations of large DNA blocks in the ACT alignment of the five strains; these integrations were found in both single strains and multiple strains. If these occurred in multiple strains at the same genomic location, the gene contents of the inserts were compared to determine if they represented distinct GIs. This process identified GIs present at 21 additional genomic locations (Table 2).

Fourth, the contents of all identified GIs were analyzed to determine if they met the criteria described above. Most identified GIs contained low %G+C, sequence composition different from the core backbone composition, and were large inserts. In addition, insertions usually were located adjacent to tRNA genes, flanking direct repeats or insertion sequence (IS) elements, or mobility genes such as integrases or transposases.

**Figure 1:**
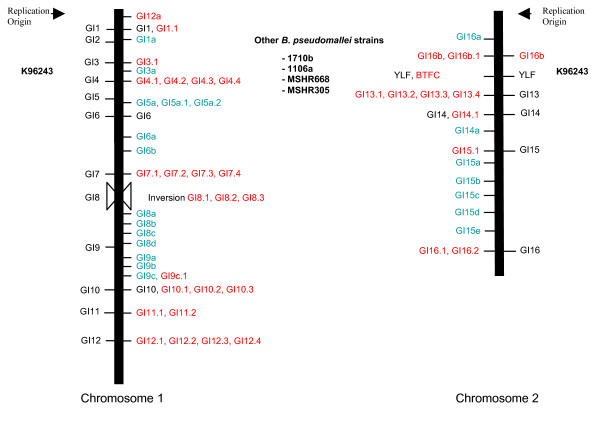
**Genomic locations of 71 GIs on chromosomes 1 and 2 in *B. pseudomallei***. GIs identified from strains 1710b, 1106a, MSHR668, and MSHR305 (inside of lines) are compared to the original 16 GIs identified from strain K96243 [11] (outside of lines). The genomic location of two mutually exclusive genomic regions, BTFC (*B. thailandensis*-like flagella and chemotaxis gene cluster) and YLF (*Yersinia*-like fimbrial gene cluster), [12] is also indicated.

GIs appear to be a major source of genomic diversity within *B. pseudomallei *as each strain has a distinct set of GIs (Table 2). We identified 71 distinct GIs among the five strains that we examined. The number of GIs identified from each strain varied: a total of 17, 16, 16, 17, and 21 GIs were identified from strains K96243, 1710b, 1106a, MSHR668, and MSHR305, respectively. For all five strains, more than half of the GIs found in that strain were unique to that particular strain. At seven of the 37 genomic locations, GIs were present in all five strains. However, none of these seven genomic locations contained the same GI across all five strains. These data illustrate that particular GIs are present in some *B. pseudomallei *strains but absent in other strains. We note that we identified a new GI in strain K96243, GI16b, which is located between GI16 and GI13 on chromosome 2 in K96243. Locations of GIs in strains 1710b, 1106a, MSHR668 and MSHR305 aligned against the relative locations of the original 16 GIs from K96243 are illustrated in Figure 1 and listed in Table 2. Sizes of the GIs ranged from 3.91 Kb for GI7.4 in MSHR305 to 107.94 Kb for GI6b in 1710b. Interestingly, dinucleotide signatures [17] 'GC' and 'CG' have the highest frequencies in all GIs regardless of the actual %G+C [see Additional file 2]. This and other information for all GIs is presented in Additional file 3.

We identified GIs in *B*. *pseudomallei *based upon the aligned differences observed across multiple genomes, which could be explained by horizontal acquisition of DNA segments from other organisms. Recently Vernikos and Parkhill [18] presented an interesting machine learning approach for identifying GIs in bacteria based upon weights of eight criteria, including: the interpolated variable order motif (IVOM) score, presence/absence of integrase, presence/absence of phage-related protein domains, size, RNA, density, repeats, and insertion points. Their studies in three different bacterial genera, *Salmonella*, *Staphylococcus*, and *Streptococcus*, suggest that GIs can be seen as a superfamily of mobile elements, with core and variable structural features, rather than a well-defined family.

#### ii) Nomenclature of *B. pseudomallei *genomic islands

Each of the five strains of *B. pseudomallei *that we examined contained unique GIs. Thus, it seems quite likely that the number of GIs discovered in this species will continue to increase as additional genome sequences become available. To facilitate scientific communication, we feel it is necessary to create a nomenclature for these important sources of genomic diversity. We propose a GI nomenclature system based upon differences in terms of locations and gene content between the newly identified GIs and the original 16 GIs identified in K96243. K96243 was chosen as the reference genome for GI locations because it was the first *B. pseudomallei *strain whole-genome sequenced and it is commonly used in many other studies. Detailed criteria for the proposed nomenclature are as follows:

##### a) Precedence

Any GIs found at the same relative GI locations and containing the same gene contents will be given the same name as the GIs previously identified in K96243. For example, strains 1710b and 1106a contain GIs at same position as GI1 of K96243 and both the GIs in these strains have similar gene composition to GI1 of K96243. Hence, these GIs also are called GI1.

##### b) Unique gene composition

Any GIs containing different gene content but located at the same reference genomic location will be given the same name as GIs in K96243 but with suffix differentiation based upon the order of their discovery (*e.g*., x.1, x.2, x.3). For example, strain MSHR668 contains a GI at the same location as GI1 in K96243 but it has different gene content. Thus, the GI in MSHR668 is designated as GI1.1, which indicates that this GI is located at the same location as GI1 but has different gene content. Some GIs may be found at the same genomic location and share highly similar sets of genes but contain differing numbers of mobility genes, suggesting different recombination events or mechanisms. These would be considered distinct GIs and given unique suffix designations. This criterion describes the difference between GI14.1 of MSHR668 and GI14 in K96243, 1710b, 1106a, and MSHR305. Both GIs contain similar genes but GI14.1 contains an extra pair of IS*407A *transposase genes, indicating that the GIs resulted from different recombination events. Using this criterion, we are able to identify different GIs that are located at the same reference location across multiple strains of *B. pseudomallei*.

##### c) Unique genomic location

Any novel GIs discovered at genomic locations located between two consecutive reference GI locations in K96243 will be named with a lower case letter (a to z) suffix indicating a new insertion position. Whenever possible these will be named in alphabetic ascending order consistent with the numerical order (*e.g*., GI9: GI9a, GI9b, GI9c: GI10). However, subsequent GI discovery studies may require a non-sequential naming, which we think is still preferable to a more complex system with additional digital codes. In this study, MSHR668 contains a new GI located between the genomic locations of GI1 and GI2 in K96243; this new GI is named GI1a. We use "a" because this is the first new GI discovered in this genomic region. We also used this rule to name GI9a, GI9b and GI9c, which are new GIs located in between the K96243 genomic positions of GI9 and G10. It is important to note that these new reference positions sometimes contain multiple GIs. In such a case, the GI name will be consistent with naming rule b and a "dot numeral" is added as a suffix (*e.g*., ".1"). In this study, we identified a new GI, GI9c, in MSHR668 but strain MSHR305 also contains a new GI at this same genomic location but with different gene content. The MSHR305 GI is therefore named GI9c.1 in accordance with naming rules b and c.

Using these three naming conventions, we are able to name new GIs discovered from multiple *B. pseudomallei *genomes according to their genomic location order, and this system should accommodate new GIs that will be discovered as new genome sequences become available.

### Genetic recombination of genomic islands

Many genomic islands in *B. pseudomallei *are created by site-specific recombination mechanisms. Site-specific recombination (SSR) involves the alignment of identical, or nearly identical sequences followed by the breaking and joining (crossover) of the strands, resulting in the exchange of genetic material. In the case of genomic islands, these recombination events are part of the integration process of foreign genetic materials. We observed at least two SSR-types, tRNA-SSR and gene specific-SSR, among the five genomes of *B. pseudomallei *examined in this study. These two types are differentiated by the recognition sequence targets used to insert the foreign genetic material.

#### i) tRNA-SSR

GIs of *B. pseudomallei *are most commonly located next to tRNA genes. There are 9, 7, 7, 10, and 10 GIs located adjacent to tRNA genes in K96243, 1710b, 1106a, MSHR668, and MSHR305, respectively (40–60% of all GIs found in these strains; see Additional file 1: Figures S1a - S1j, and Table 3). In *B. pseudomallei *genomes, there are between 59 to 61 tRNA genes distributed across both chromosomes. Recombination at tRNA loci is initiated at the 3' end of tRNA genes. This process creates a short, direct repeat sequence of the tRNA gene downstream of the integration site. We termed this type of recombination "tRNA-mediated site-specific recombination" or "tRNA-SSR". The genomic recognition sites are as short as a 14 bp repeat sequence (tRNA-Ser; GI13) and as long as a 56 bp repeat sequence (tRNA-Leu; GI5). Table 3 summarizes the locations of all 3'end repeats of tRNA genes that are associated with GIs in the five *B. pseudomallei *genomes. We noted that not all GIs located next to tRNA genes contain the short direct repeat sequences (Table 3). This suggests that multiple recombination events at the same recognition site may have caused the repeat sequences to disappear. Details of tRNA sequences and their repeat sequences are presented in Additional file 4: Tables S2.1-S2.5. Numbers of tRNA-SSR are varied among the five *B. pseudomallei *strains. At least 8, 3, 2, 5, and 5 tRNA-SSR events are present in K96243, 1710b, 1106a, MSHR668, and MSHR305, respectively. In addition, recombination events at tRNA-Met, Pro, Thr, Ala, and Arg are common across the genomes (Table 3), suggesting that these sites serve as "genomic hotspots" for GI insertion in *B. pseudomallei *genomes.

**Figure 2:**
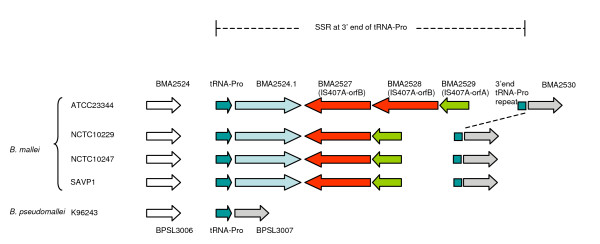
**Site-specific recombination (SSR) at tRNA-Pro in *B. mallei *causes a 3' end repeat 26 bp downstream of the recombination site**. This site contains insertion element *IS407A*, which is common in *B. mallei *genomes. This suggested that *IS407A *was present in other mobile genetic elements when they were originally introduced to *B. mallei *genomes and the recombination event was associated with tRNA-Pro.

**Figure 3:**
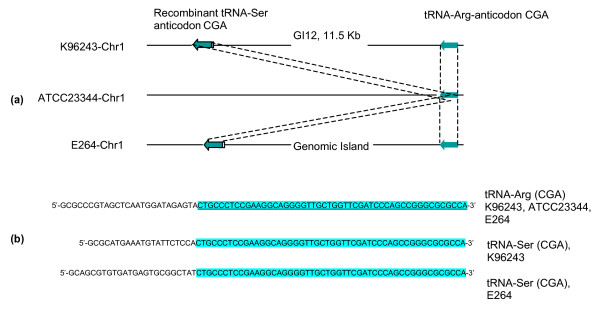
**Predicted site-specific recombination (SSR) events at tRNA-Arg (CGA) in *B. pseudomallei *K96243 and *B. thailandensis *E264 and the relative genomic region in *B. mallei *ATCC23344**. (a) An extra recombinant tRNA-Ser (CGA) was created in the genomes of these two bacterial strains by SSR; parallel dash lines indicate identical regions of the tRNA genes. (b) Nucleotide sequences of the tRNA-Arg and the recombinant tRNA-Ser; the predicted SSR-recognition site of tRNA-Arg in strains K96243 and E264 is underlined, and the identical regions between these two tRNA genes are highlighted in blue.

To evaluate whether this type of genetic recombination is present in other *Burkholderia *species, we performed similar analysis on four *B. mallei *genomes, strains ATCC23344 [19] (accession no. CP000010&CP000011), NCTC10247 (accession no. CP000547&CP000548), NCTC10229 (accession no. CP000545&CP000546), and SAVP1 (accession no. CP000525&CP000526); as well as the genome of *B. thailandensis *strain E264 (accession no. CP000085&CP000086). Similar to *B. pseudomallei*, tRNA-SSR events at tRNA-Pro genes were found in all four *B. mallei *strains. In addition, these integration events were all mediated by a well-known insertion element, IS*407A *[19] (Figure 2). In *B. thailandensis *E264, Yu and colleagues [20] previously identified 15 different genomic islands, known as Bt-GI1 to Bt-GI15. We aligned all 15 GIs from this *B. thailandensis *strain to the relative genomic locations of GIs in *B. pseudomallei*. In *B. thailandensis *E264, we found one tRNA-SSR event present at tRNA-Ser and two at tRNA-Arg, corresponding to the same genomic locations as GI13, GI7.1, and GI12 in *B. pseudomallei*, respectively [see Additional file 5]. In addition, the 3'end repeat downstream of tRNA-Arg in *B. pseudomallei *K96243 (GI12) and *B. thailandensis *E264 has caused a recombinant tRNA-Ser. This is an unusual event in *B. pseudomallei *genomes and it is not known if this extra tRNA-Ser is functional in these *B. pseudomallei *and *B. thailandensis *strains. Figure 3 illustrates the recombination events that we propose caused these new recombinant tRNAs-Ser in *B. pseudomallei *K96243 and *B. thailandensis *E264. Details of all tRNA-SSR events and 3'end tRNA repeat sequences in the *B. thailandensis *and *B. mallei *genomes are presented in Additional file 4: Tables S2.6 - S2.10.

#### ii) Gene specific recombination

**Figure 4:**
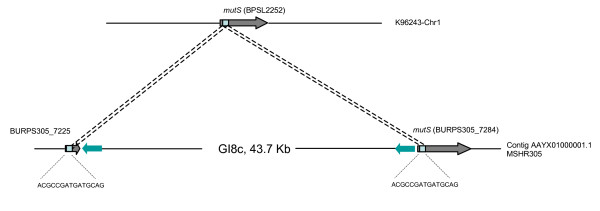
**Site-specific recombination at the *mutS *gene of *B. pseudomallei *MSHR305**. This event caused a short direct repeat 15 bp downstream of GI8c. GI8c is a putative prophage of MSHR305.

We identified a site-specific recombination event at the *mutS *gene (DNA mismatch repair protein) in strain MSHR305 that is associated with GI8c of *B. pseudomallei*. The insertion of GI8c caused two direct repeats of 15 bp located on genes BURPS305_7225 (DNA mismatch repair protein) and BURPS305_7284 (*mutS*, DNA mismatch repair protein). The genes have different sizes and are only partially identical. A 15 bp recognition site, 5'-ACGCCGATGATGCAG-3', of the BURPS305_7225 gene is predicted to be involved in this genetic recombination event (Figure 4). Additionally, we found that recombination at the *mutS *gene was also associated with Bt-GI4 in *B. thailandensis *E264. To our knowledge, this *mutS*-specific recombination is reported here for the first time.

Recognition sites at the 3'end of various tRNA genes and *mutS *genes in *B. pseudomallei *and *B. thailandensis *serve as hotspots for genetic recombination in these bacterial species. tRNA-SSR has been reported in other Gram-negative bacteria such as the VPI-2 pathogenicity island of *Vibrio cholerae*, which is associated with the sequence of tRNA-Ser [21]. Recombination at a specific gene also has been reported at the *glr *(glutamate racemase) gene of *Helicobacter pylori *where it is served as the recognition or integration site of the cag pathogenicity islands [22].

### Gene contents and predicted functional roles of GIs

Overall, most GIs contain genes known to be involved with genomic mobility and genetic recombination. These genes include transposase, integrase, conjugal plasmid protein, recombinase, invertase, and resolvase genes. More than 80% of GIs contain at least one transposase genes. Transposase genes are known as major components of insertion elements in bacterial genomes. Most common IS elements located in the genomic islands are members of the IS*3 *family, including IS*407A *and IS*Bp1 *[23]. IS*407A *was found in most GIs from all strains except MSHR305, whereas IS*Bp1 *was only found in GIs of strains 1710b and 1106a. MSHR305 contains unique IS elements in its GIs, such as IS*Psy16*, IS*Afe1*, IS*Afe4*, and IS*rso12*, that are uncommon in GIs from other strains. This suggests that GIs in *B. pseudomallei *originated from different sources, as they were brought to the species by different insertion elements.

**Figure 5:**
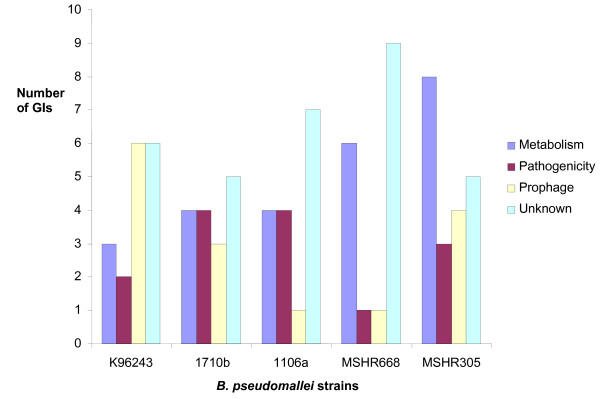
**Prevalence of GIs with four predicted functions in five reference *B. pseudomallei *strains**. Interestingly, strains MSHR668 and MSHR305 from Australia contain a high number of GIs associated with metabolism.

To predict functional roles of GIs in *B. pseudomallei*, we classified all GIs from the five *B. pseudomallei *strains into four different functional categories: prophage, metabolism, pathogenicity, and unknown. GIs can contain genes that are predicted to be involved in more than one of these functions. In this study, we classified GIs based upon the functional role of a majority of the genes. Abundance of genomic islands for these four major categories in five strains of *B. pseudomallei *is shown in Figure 5. Details of all 4 categories are as follows:

#### i) Prophages

Many GIs in *B. pseudomallei *are prophages or prophage-like structures. The first functional prophage in *B. pseudomallei *was identified in K96243 [11]. Following UV induction, strain K96243 produced at least one lysogenic phage known as ΦK96243, which was able to infect *B. mallei*. DNA sequence analysis of ΦK96243 indicates that it is associated with GI2, which contains approximately 36.3 Kb of genes necessary for bacteriophage biogenesis. We note that GI2 is located directly downstream of tRNA-Phe and contains a direct repeat (45 bp) of the 3' end sequence of tRNA-Phe. This repeat sequence is believed to be generated during bacteriophage lysogenization at the recognition site, known as *att*B, which is the 3' end sequence of tRNA-Phe. GI2 is not present in the other four *B. pseudomallei *genomes examined here, but it is found in other *B. pseudomallei *strains such as Pasteur 52237 (accession no.AAHV00000000) and S13 (accession no. AAHW00000000) (data not shown).

Another well characterized prophage in *Burkholderia *species is associated with site-specific recombination at the 3'end of tRNA-Pro in *B. thailandensis *E125. ΦE125, a temperate bacteriophage from *B. thailandensis*, has been shown to insert as a lysogen in *B. mallei *ATCC23344 by site-specific recombination at the 3'end of tRNA-Pro [24]. This type of prophage in *B. pseudomallei *was firstly described in strain 1026b [25].

Bacteriophage induction occurred spontaneously during normal growth of strain 1026b in liquid culture. We found that strain 1710b contains a prophage (GI6b) that is similar to the functional prophage of 1026b, but it is two-fold larger in size. Both prophages are similar since they are associated with tRNA-SSR at tRNA-Pro. Surprisingly, GI6b contains two 3'end tRNA-Pro repeats (49 and 23 bp; Additional file 4: Table S2.2), suggesting multiple site-specific recombination events. There are no data demonstrating that GI6b in strain 1710b is a functional prophage or can be induced. However, this putative prophage does contain genes for bacteriophage biogenesis and also a putative phospholipase gene (BURPS1710b_1675), suggesting that specialized transduction has occurred. Several studies have shown that phospholipase genes are potential virulence genes in *B. pseudomallei *[26,27].

Site-specific recombination that forms bacterial prophages is not only associated with specific tRNA-genes, but also with specific gene targets. MSHR305 contains a putative prophage in GI8c that is associated with the *mutS *gene. Recombination has created a small 15 bp repeat downstream of the genomic island, as described earlier. We also note that strain 1106a contains only one prophage (GI10.2) in its genome, which is associated with site-specific recombination at tRNA-Thr. Again, there are no data confirming that the GI10.2 is a functional prophage, although its structure does contain bacteriophage biogenesis genes.

#### ii) Metabolism

GIs containing metabolic genes are variable across different *B. pseudomallei *strains and populations, which may affect bacterial fitness and be related to specific environmental niches. Nineteen of the 71 (26.7%) GIs we identified in *B. pseudomallei *contain metabolic genes [see Additional file 3]. GI14 and GI14.1 are similar in term of gene contents and both contain genes with predicted metabolic functions. Hence, both represent potential metabolic islands. The genes in these islands include peptidase enzymes such as collagenase (BPSS0666), alpha-ketoglutarate-dependent taurine dioxygenase (BPSS0665), and x-prolyl-dipeptidyl aminopeptidase (BPSS0654). These genes are predicted to be involved in amino acid metabolism but there are no studies on their actual functional roles. We used a PCR assay to query the presence/absence of the BPSS0654 taurine dioxygenase gene across large *B. pseudomallei *strain collections from Thailand and Australia. We found that this gene is present in 99% and 97% of tested strains from these two countries, respectively [see Additional file 6]. We note that strains MSHR668 and MSHR305 contain six and eight GIs with metabolic genes, respectively (Figure 5). Metabolic GI gene BURPS305_5421 (bacterial extracellular solute-binding protein) in MSHR305 has a very limited distribution, occurring in less than 10% of tested strains from Thailand and 56% of tested strains from Australia [see Additional file 6].

#### iii) Pathogenicity

We have identified two different groups of genes in *B. pseudomallei *GIs that could have functional roles in bacterial pathogenicity, especially bacterial adherence. They are genes that encode for filamentous hemagglutinin proteins (FHA) and two-partner secretion systems (TPS).

**Figure 6:**
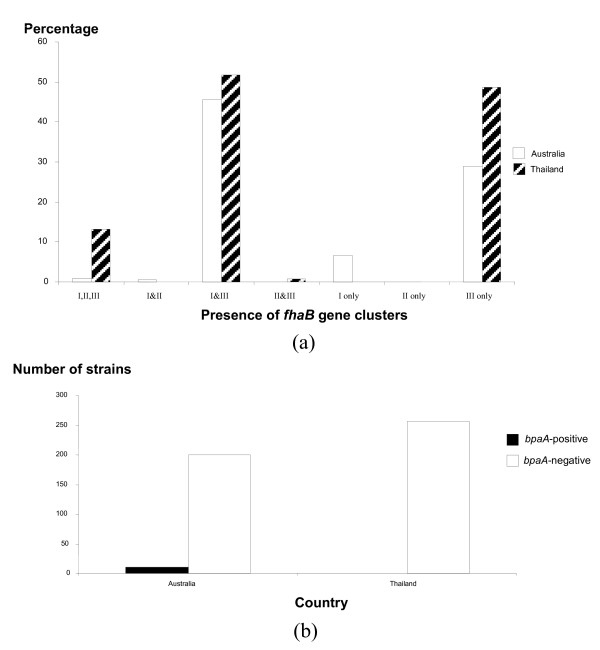
**Distribution (presence) of GI genes within diverse *B. pseudomallei *populations**. (a) Presence of *fhaB *gene clusters. (b) Presence of *bpaA *gene. We note that most *B. pseudomallei *strains contained either two clusters of the *fhaB *gene, clusters I and III, or cluster III alone. In addition, the *bpaA *gene was found only in clinical isolates of *B. pseudomallei *from Australia.

FHA genes in *B. pseudomallei *are located on different genomic islands and varied among strains. Strain 1106a contains three different clusters of *fha *genes located at GI5a.1, GI11.1, and GI16.1; strain 1710b contains two clusters (GI5a and GI16.1); strain K96243 contains one cluster at GI16; and strains MSHR668 and MSHR305 contain only one cluster at GI5a.1 and GI5a.2, respectively. Homologs of *fha *genes have been reported in other Gram-negative bacteria where they have functional roles in virulence. When we compared the *fhaB *gene, which is the largest gene in each *fha *gene cluster, to its homolog in *Bordetella pertussis*, we found high similarity to all three *B. pseudomallei *gene clusters. Thus, it seems likely that all three genes encode a filamentous hemagglutinin domain protein in *B. pseudomallei *[see Additional file 7]. More recently, Sim and Yu *et al*. reported that the experimental mutation one *fhaB *gene (BPSS2053, equivalent to *fhaB3 *gene in our study) in *B. pseudomallei *DD503 caused the reduction of microbial adherence to human epithelial cells [28]. We also examined the occurrence of this gene among diverse *B. pseudomallei *populations and found that *fhaB *copy number varied among the strains. Most *B. pseudomallei *strains from Thailand and Australia contained either two clusters of *fhaB *genes (cluster I from GI5a/GI5a.1/GI5a.2 and cluster III from GI16/GI16.1), or a *fhaB *gene from cluster III (GI16/GI16.1) alone. In contrast, cluster II found in strain 1106a (GI11.1) was very rare. Details of the distribution of *fhaB *gene clusters in *B. pseudomallei *are demonstrated in Figure 6a and a list of tested strains is provided in Additional file 6.

Two-partner secretion systems in *B. pseudomallei *were first described by Brown and colleagues in 2002 [29]. They reported that this particular two-partner secretion system was encoded by genes *bpaAB*, which were located in the GIs of strain 08 but absent in many other *B. pseudomallei *strains used in their study. We have determined that these genes are located in GI15d of MSHR305 and not observed in the other four *B. pseudomallei *genome sequences examined here. We examined the frequency of the *bpaA *gene (the largest gene in GI15d) among diverse *B. pseudomallei *strains and found that it was rare. Only 2.3% of strains contained this gene and, interestingly, these strains were only found in clinical isolates from Australia [see Figure 6b; Additional file 6]. *B. pseudomallei *virulence determinants are not well understood, but animal models have demonstrated that different strains do differ in their virulence levels [30,31] and this could be related to variation in GIs and GI content.

#### iv) Unknown functional roles

The functional roles of many genomic islands in *B. pseudomallei *(32%) is unknown; most genes found in *B. pseudomallei *GIs have only hypothetical protein predictions that do not match any characterized proteins databases.

The four categories of functional roles of GIs described above are based primarily upon computer predictions and only a minimal number of laboratory studies. Direct functional studies of GI genes are very rare, and most functional genomics studies rely upon *B. pseudomallei *gene conservation compared to other species. Almost all comparative genomic studies among *B. pseudomallei *strains identify differences primarily among genomic islands genes [25,32,33]. In a 2006 study by Duangsonk and colleagues [32], GI genes identified by suppression subtractive hybridization (SSH) between two different *B. pseudomallei *strains were used to develop a variable amplicon typing (VAT) scheme. VAT was applied successfully to population genetics problems and differentiated groups of *B. pseudomallei *strains that caused different clinical outcomes. Most recently, genes of five GIs from K96243, including GI2, GI6, GI9, GI11, and GI16, were used to measure frequency values among clinical and environmental *B. pseudomallei *strains from Thailand [34]. There were no significant differences between environmental and disease-associated isolates for these genes. However, because only GIs from K96243 were used, this study was not representative of the large GI diversity observed among multiple *B. pseudomallei *genomes. Still, this is one of the first examples attempting to correlate these very important genomic components to clinically relevant phenotypes. Because of the importance of GIs in the accessory genome of *B. pseudomallei*, they may play important roles in various phenotypic differences within the *B. pseudomallei *species. Studies of GI genes in various fields of research, such as population genetics and functional genomics, warrant further investigation but this must be based upon pan-genomic analysis using all available data.

## Conclusion

The data presented here suggest that a large number of GIs have been acquired by horizontal acquisitions and that these GIs represent a major source of genomic diversity in *B. pseudomallei*. The proposed nomenclature suggested above will be important for effective communication in the research community and for cataloging the highly variable GIs. tRNA-mediated site-specific recombination appears to be an important mechanism for horizontal gene transfers of GIs. The differential presence of GIs in multiple strains also demonstrates the limited phylogenetic distribution of mobile genetic elements in this bacterial species. GIs are part of the accessory genome, which have not been studied thoroughly in *B. pseudomallei*. Various fields of post-genomics, such as population genetics and functional genomics of GIs, are worthy of further investigations.

## Methods

### Genomic Data

Genomes of five clinical *B. pseudomallei *strains were used in this study. These strains included K96243, 1710b, 1106a, MSHR668, and MSHR305. Clinical information and genomic features of all five strains are summarized in Table 1.

### Comparative genomics, genome alignment, and bioinformatics of genomic island

Comparisons of the genome sequences for most aspects of this study were performed using ACT (Artemis comparison tool) [15]. Each genomic comparison file was generated from NCBI- Local BLAST program, which is available in BioEdit Sequence Alignment Editor [35]. Dinucleotide signatures among all genomic islands were analyzed using an in-house Java program. Dinucleotide frequencies and indices were calculated according to Karlin and Burge [17]. Circular diagrams of all genomes used in this study were made by using CGView [36].

### Experimental examination of genomic islands

SYBR-Green real-time PCR assays were developed to examine the presence/absence of genomic island genes across multiple strains of *B. pseudomallei*. Specific genes from six genomic islands were selected and used as the targets for PCR. These included three different clusters of *fhaB *genes located in several different GIs; *bpaA*, a known gene encoding for a two-partner secretion system; and two metabolic genes, BPSS0654 of GI14 and BURPS305_5421 of GI14a. Details of PCR primers and target genes are described in Additional file 8. We used genomic DNA samples from five genome-sequenced strains, K96243, 1710b, 1106a, MSHR668 and MSHR305 as positive and/or negative PCR controls. A total of 468 genomic DNA samples were used in the analysis.

## Authors' contributions

AT conceived of the study, performed major analyses, and drafted the manuscript. BRL performed validation of most experiments. RKA, SMBS, and JMBS provided bioinformatic assistances and analyses. MM and VW provided DNA samples for testing throughout the study. TSB provided shotgun genome sequences of MSHR305. WCN, SJP, BJC, DMW and PK participated in the design and coordination of the study and manuscript preparation. All authors have read and approved the manuscript.

## Supplementary Material

Additional file 1 **Circular diagrams**. Figure S1a. Circular diagram of the artificial chromosome 1 of MSHR305. Figure S1b. Circular diagram of the artificial chromosome 2 of MSHR305. Figure S1c. Circular diagram of genomic islands on chromosome 1 of K96243. Figure S1d. Circular diagram of genomic islands on chromosome 2 of K96243. Figure S1e. Circular diagram of genomic islands on chromosome 1 of 1710b. Figure S1f. Circular diagram of genomic islands on chromosome 2 of 1710b. Figure S1g. Circular diagram of genomic islands on chromosome 1 of 1106a. Figure S1h. Circular diagram of genomic islands on chromosome 2 of 1106a. Figure S1i. Circular diagram of genomic islands on chromosome 1 of MSHR668. Figure S1j. Circular diagram of genomic islands on chromosome 2 of MSHR668 Click here for file

Additional file 2 **Dinucleotide frequency**. Figure S2. Analysis of dinucleotide frequencies of all genomic islands compared to the conserved regions in *B. pseudomallei *genomes. Frequencies of dinucletides "CG and GC" are relatively high regardless of the total %G+C of the GIs. Click here for file

Additional file 3 **CDS coordinates of genomic islands**. Table S1. Details of all genomic islands described in this study. Click here for file

Additional file 4 **tRNA-SSR**. Table S2.1 tRNA-SSR distribution in *B. pseudomallei *K96243. Table S2.2 tRNA-SSR distribution in *B. pseudomallei *1710b. Table S2.3 tRNA-SSR distribution in *B. pseudomallei *1106a. Table S2.4 tRNA-SSR distribution in *B. pseudomallei *MSHR668. Table S2.5 tRNA-SSR distribution in *B. pseudomallei *MSHR305. Table S2.6 tRNA-SSR distribution in *B. thailandensis *E246. Table S2.7 tRNA-SSR distribution in *B. mallei *ATCC23344. Table S2.8 tRNA-SSR distribution in *B. mallei *NCTC10247. Table S2.9 tRNA-SSR distribution in *B. mallei *NCTC10229. Table S2.10 tRNA-SSR distribution in *B. mallei *SAVP1. Click here for file

Additional file 5 **Relative genomic island locations in *B. pseudomallei *and *B. thailandensis***. Table S3. Relative genomic island locations in 5 *B. pseudomallei *strains and *B. thailandensis *E264. Click here for file

Additional file 6 **Distribution of GIs**. Table S4. Distribution of selected genomic island genes in *B. pseudomallei *strains. Click here for file

Additional file 7 **Amino acid sequences of *fhaB *genes**. Figure S3. Showing homology of amino acid sequences of 3 *fhaB *genes in *B. pseudomallei *and a reference amino acid sequence (IRWR_chainA) for filamentous hemagglutinin of *Bordetella pertussis*. This suggests that three *fhaB *genes of *B. pseudomallei *may have the same functional role as the filamentous hemagglutinin, a virulence effector molecule in *B. pertussis*. Click here for file

Additional file 8 **PCR primers**.Table S5. PCR primers used in this study. Click here for file
