# Supplementary material for: Genomic islands from five strains of Burkholderia pseudomallei
Source: BMC Genomics. 2008 Nov 27;9:566. doi: 10.1186/1471-2164-9-566 (PMC2612704; doi:10.1186/1471-2164-9-566)

Figure S1a. Circular diagram of the artificial chromosome 1 of MSHR305


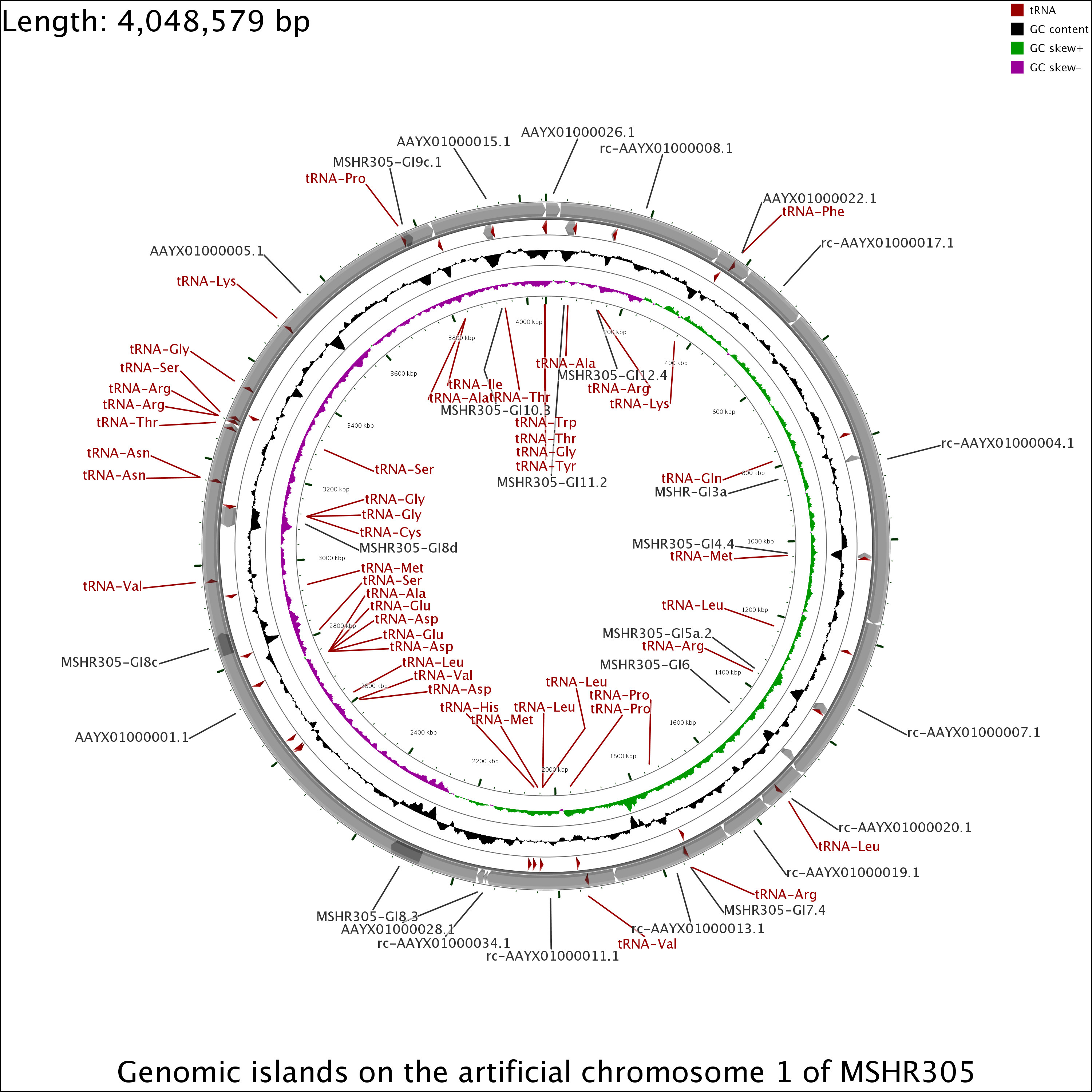


Figure S1b. Circular diagram of the artificial chromosome 2 of MSHR305


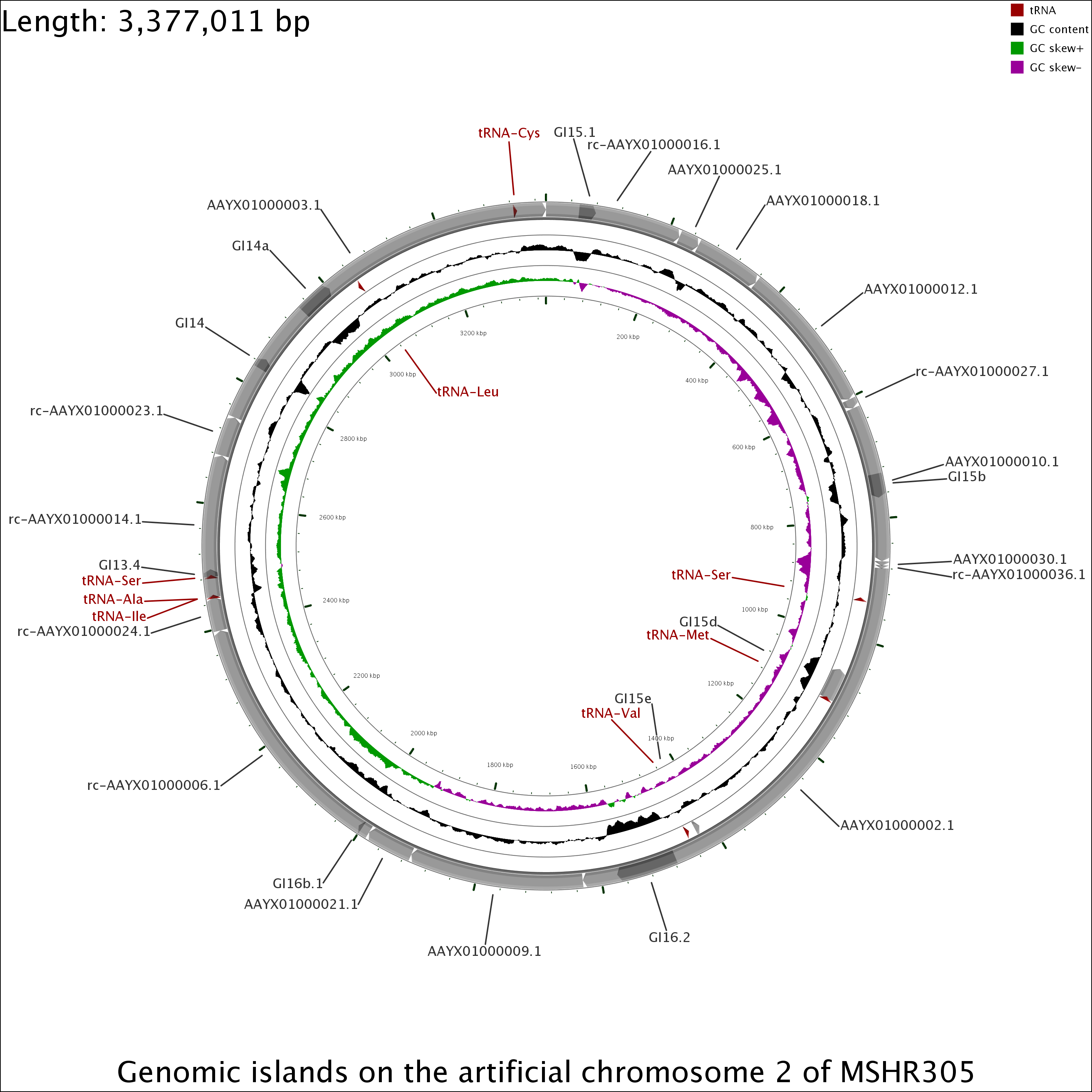


Figure S1c. Circular diagram of genomic islands on chromosome 1 of K96243


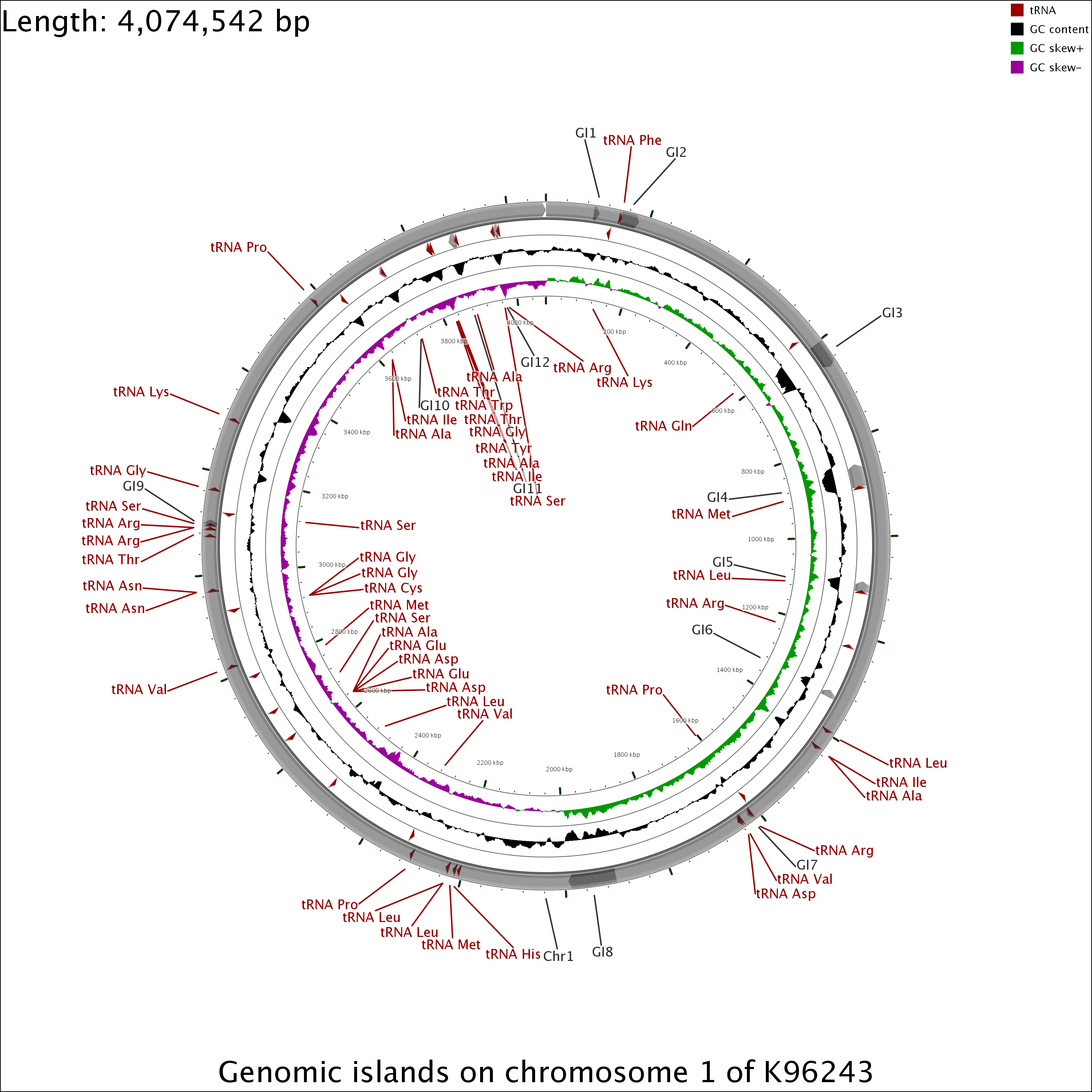


Figure S1d. Circular diagram of genomic islands on chromosome 2 of K96243


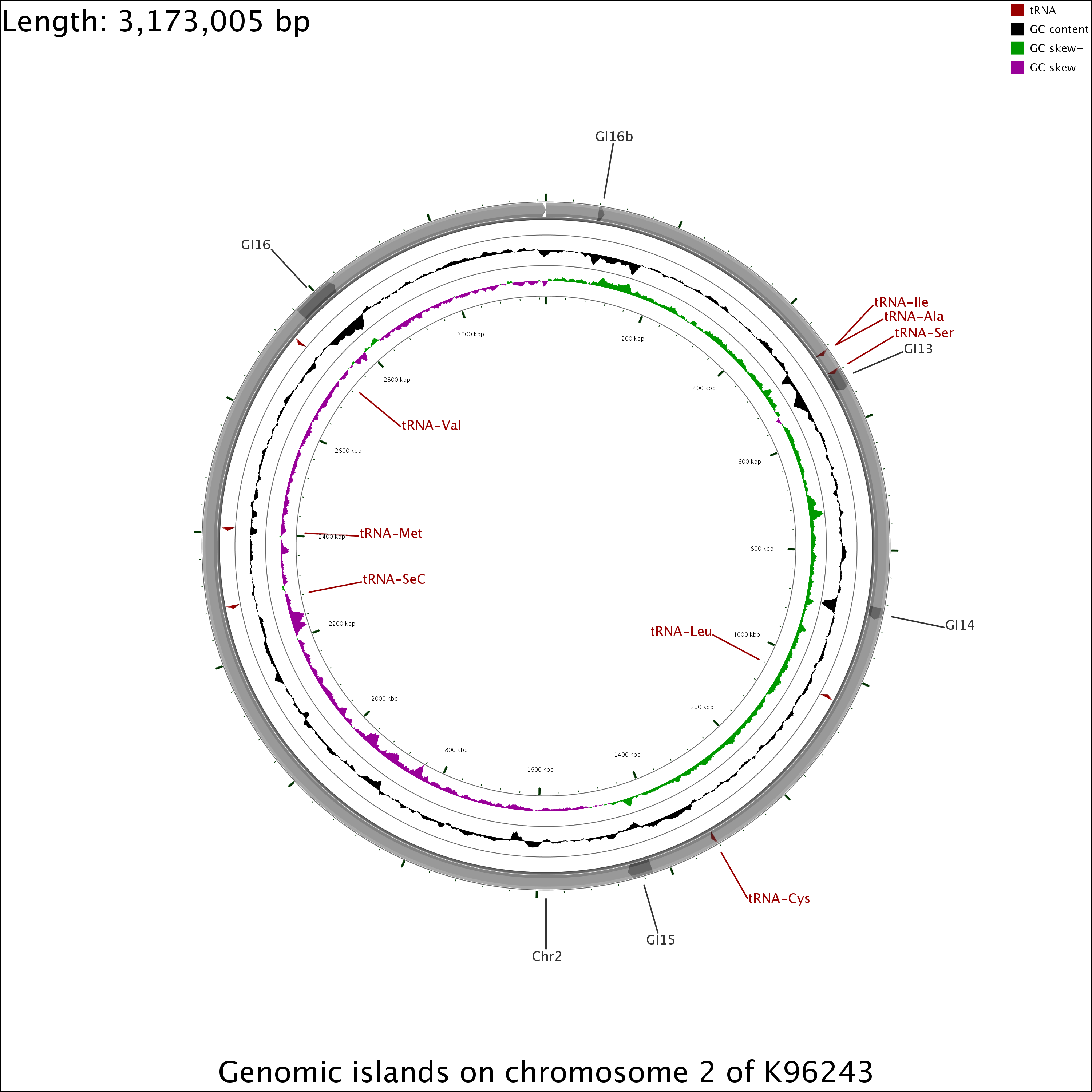


Figure S1e. Circular diagram of genomic islands on chromosome 1 of 1710b


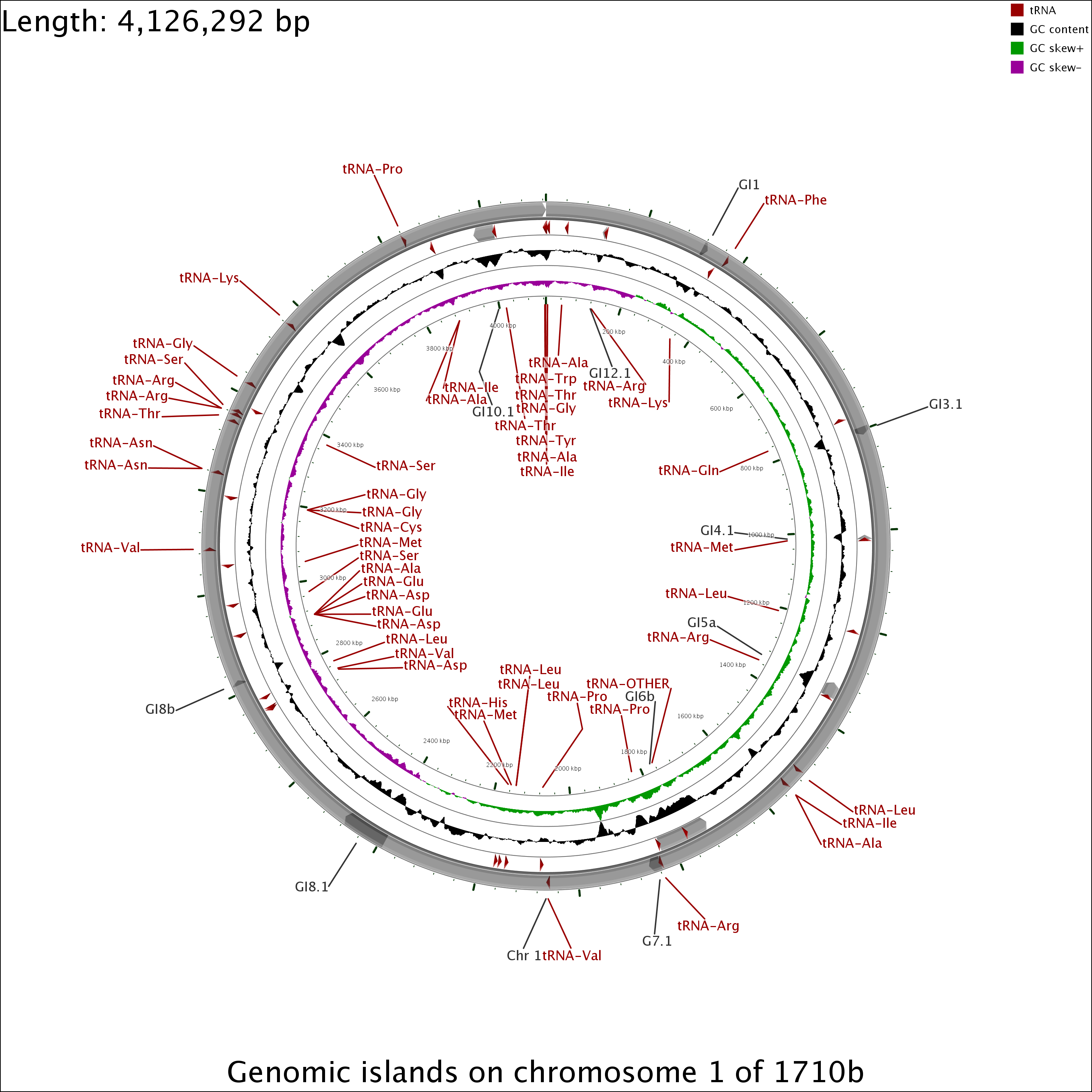


Figure S1f. Circular diagram of genomic islands on chromosome 2 of 1710b


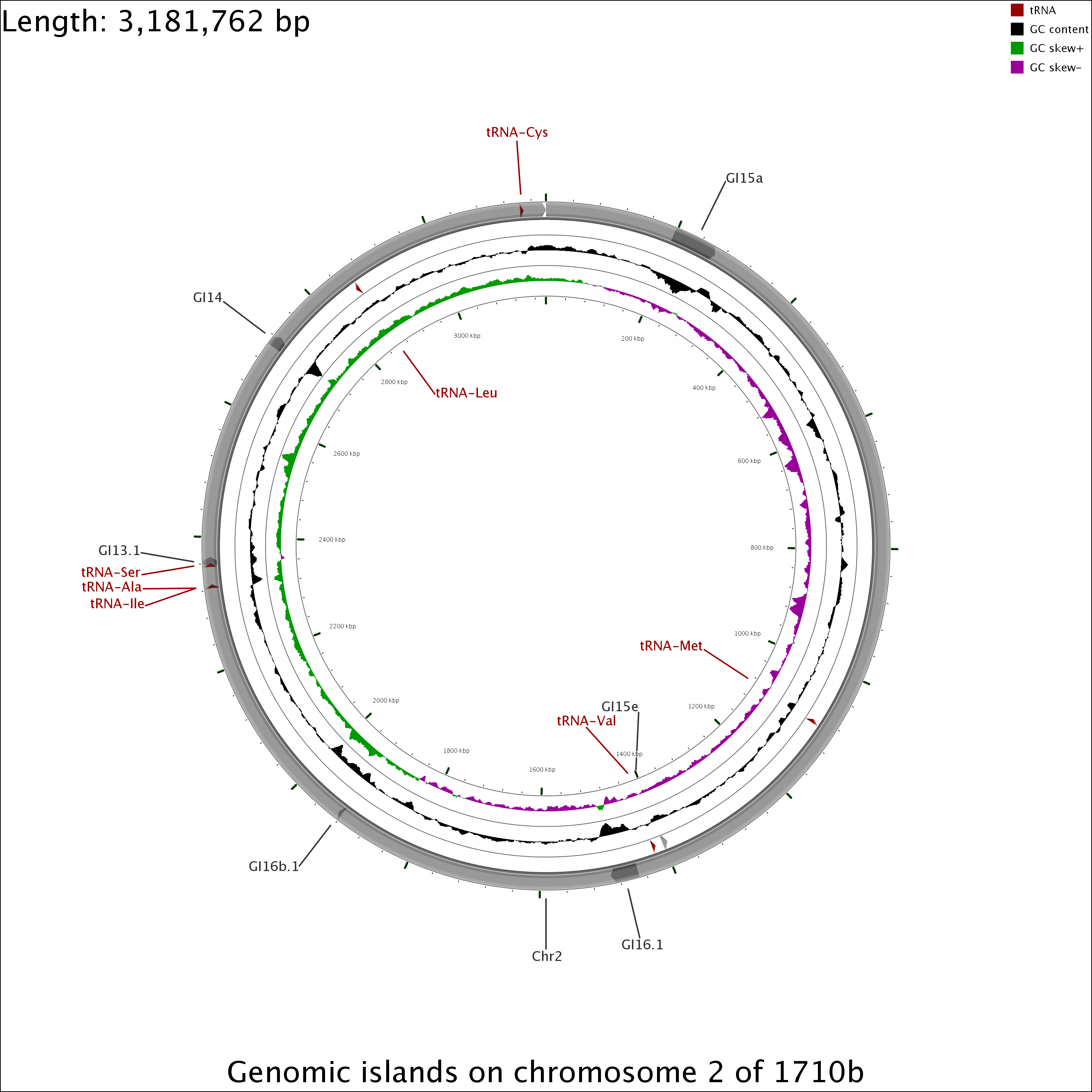


Figure S1g. Circular diagram of genomic islands on chromosome 1 of 1106a


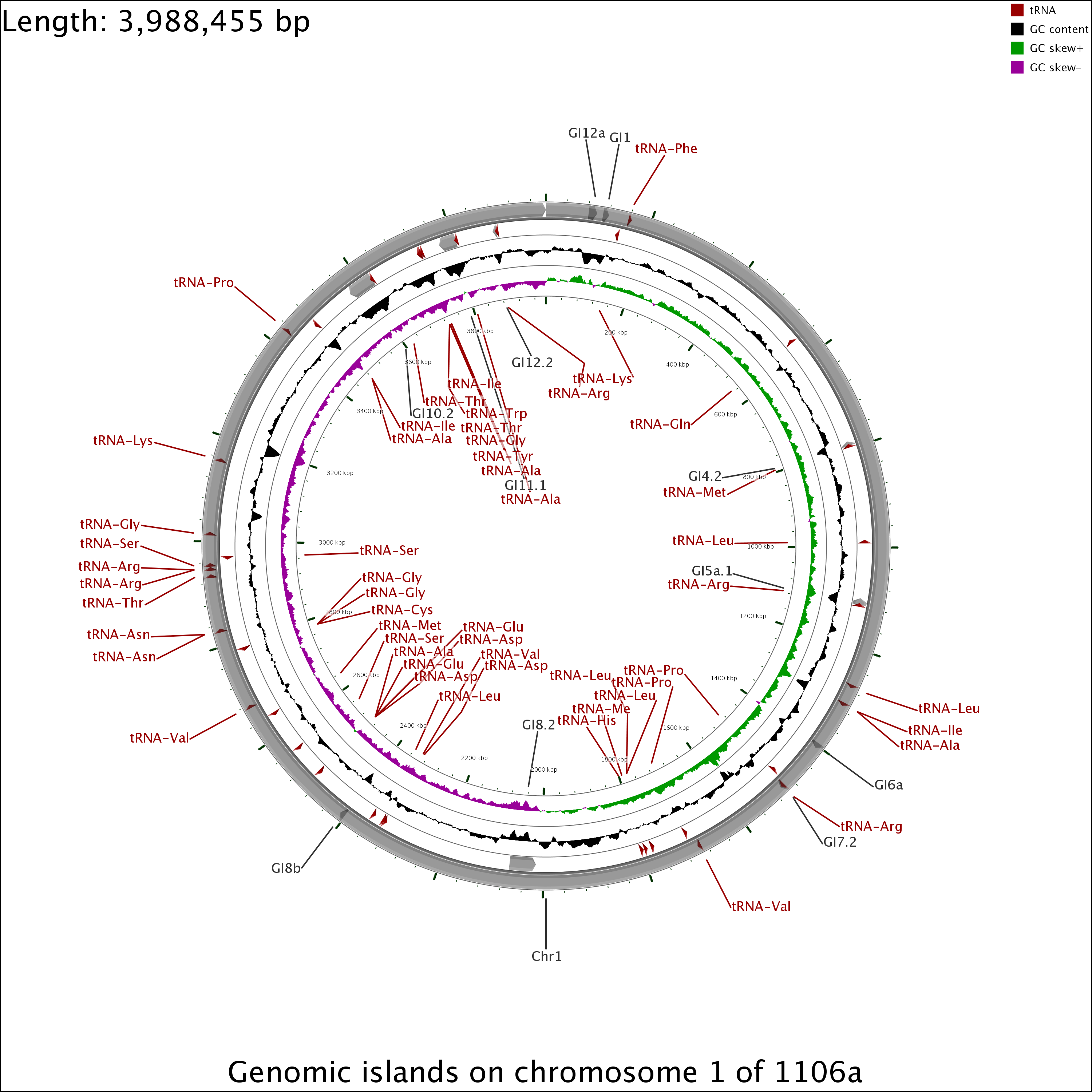


Figure S1h. Circular diagram of genomic islands on chromosome 2 of 1106a


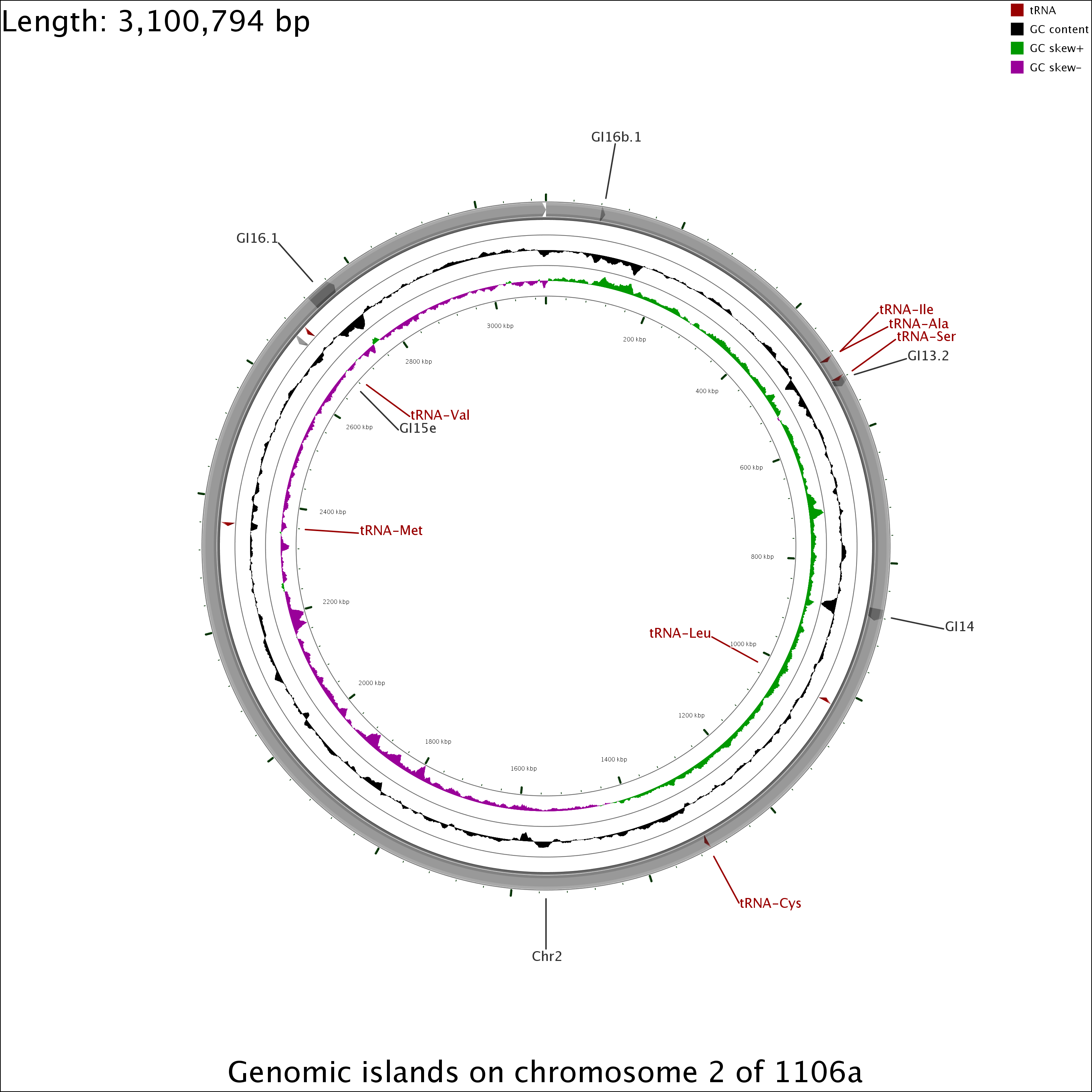


Figure S1i. Circular diagram of genomic islands on chromosome 1 of MSHR668


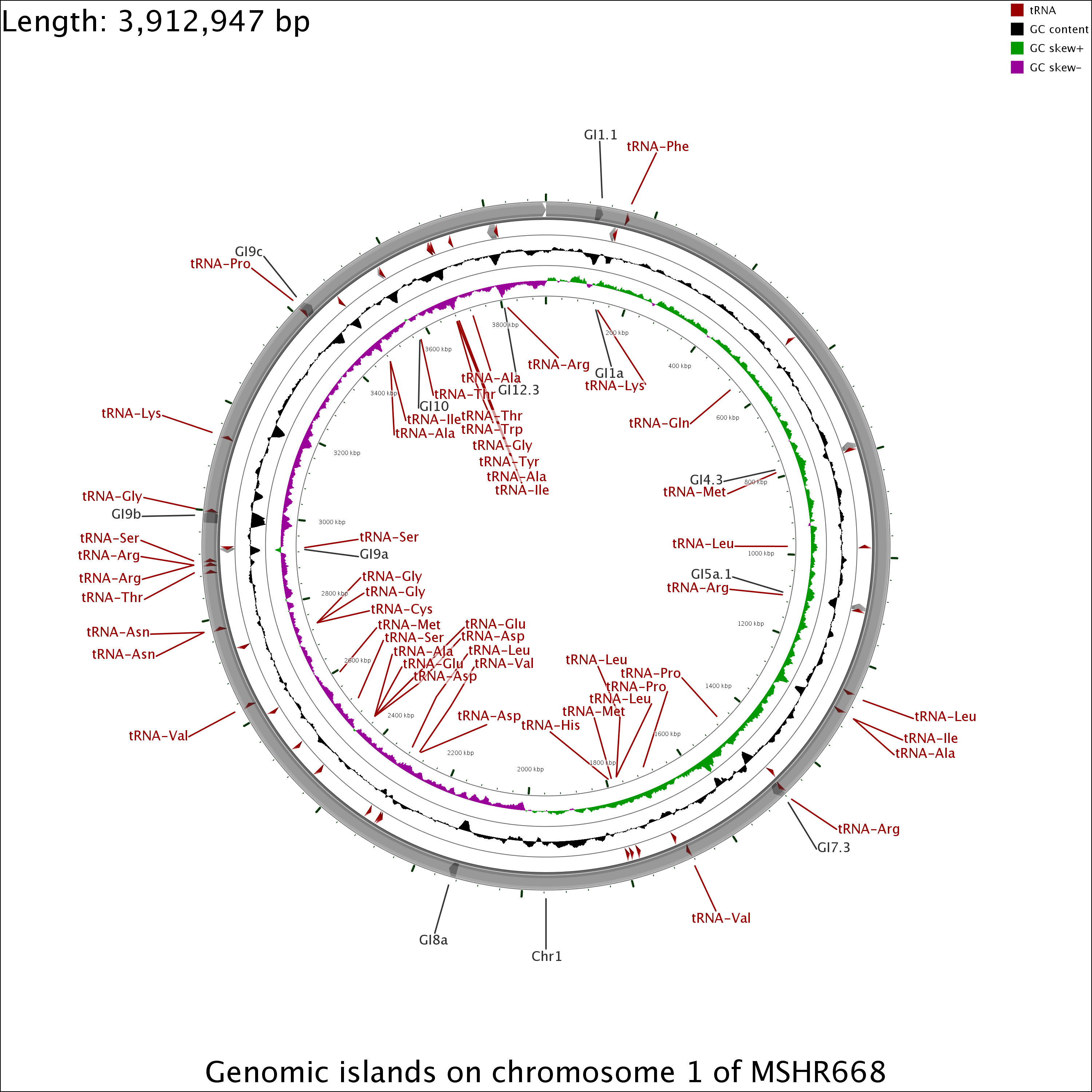


Figure S1j. Circular diagram of genomic islands on chromosome 2 of MSHR668


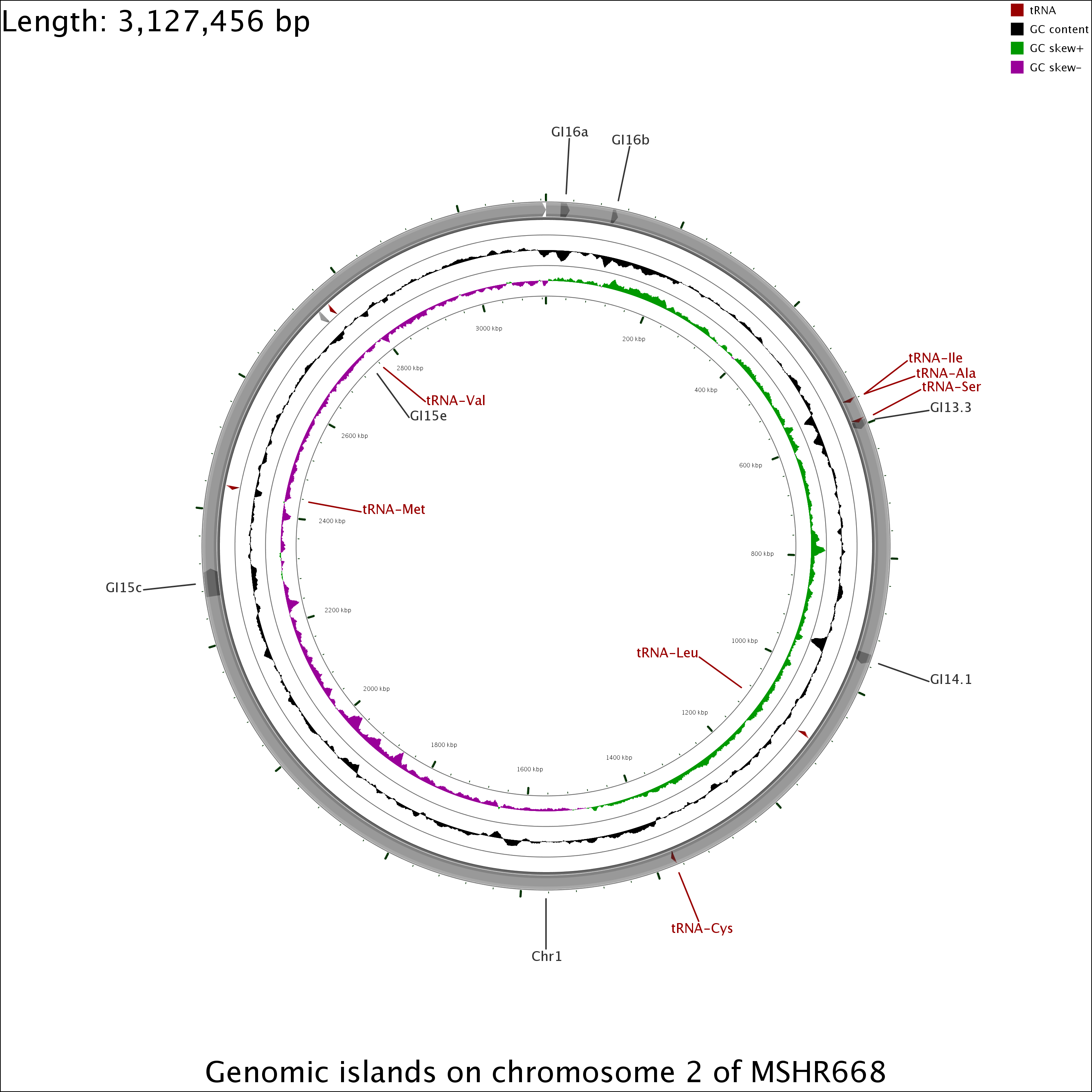

Supplement: Additional file 1 — Circular diagrams. Figure S1a. Circular diagram of the artificial chromosome 1 of MSHR305. Figure S1b. Circular diagram of the artificial chromosome 2 of MSHR305. Figure S1c. Circular diagram of genomic islands on chromosome 1 of K96243. Figure S1d. Circular diagram of genomic islands on chromosome 2 of K96243. Figure S1e. Circular diagram of genomic islands on chromosome 1 of 1710b. Figure S1f. Circular diagram of genomic islands on chromosome 2 of 1710b. Figure S1g. Circular diagram of genomic islands on chromosome 1 of 1106a. Figure S1h. Circular diagram of genomic islands on chromosome 2 of 1106a. Figure S1i. Circular diagram of genomic islands on chromosome 1 of MSHR668. Figure S1j. Circular diagram of genomic islands on chromosome 2 of MSHR668 [file 1471-2164-9-566-S1.doc]
