# Supplementary material for: Genomic islands from five strains of Burkholderia pseudomallei
Source: BMC Genomics. 2008 Nov 27;9:566. doi: 10.1186/1471-2164-9-566 (PMC2612704; doi:10.1186/1471-2164-9-566)
Supplement: Additional file 2 — Dinucleotide frequency. Figure S2. Analysis of dinucleotide frequencies of all genomic islands compared to the conserved regions in B. pseudomallei genomes. Frequencies of dinucletides "CG and GC" are relatively high regardless of the total %G+C of the GIs. [file 1471-2164-9-566-S2.doc]

Figure S2. Analysis of dinucleotide frequencies of all genomic islands compared to the conserved regions in *B. pseudomallei* genomes. Frequencies of dinucletides “CG and GC” are relatively high regardless of the total %G+C of the GIs.

GIs

Randomly picked non-GIs

Dinucleotide frequency
