# Supplementary material for: Genomic islands from five strains of Burkholderia pseudomallei
Source: BMC Genomics. 2008 Nov 27;9:566. doi: 10.1186/1471-2164-9-566 (PMC2612704; doi:10.1186/1471-2164-9-566)

Figure S3: Showing homology of amino acid sequences of 3 *fhaB* genes in *B. pseudomallei* and a reference amino acid sequence (IRWR_chainA) for filamentous hemagglutinin of *Bordetella pertussis.* This suggests that three *fhaB* genes of *B.* *pseudomallei* may have the same functional role as the filamentous hemagglutinin, a virulence effector molecule in *B. pertussis*.


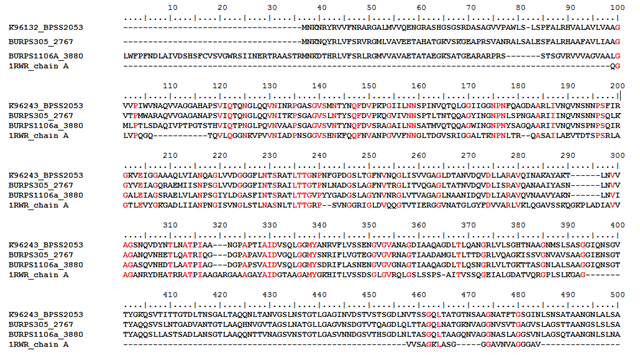

Supplement: Additional file 7 — Amino acid sequences of fhaB genes. Figure S3. Showing homology of amino acid sequences of 3 fhaB genes in B. pseudomallei and a reference amino acid sequence (IRWR_chainA) for filamentous hemagglutinin of Bordetella pertussis. This suggests that three fhaB genes of B. pseudomallei may have the same functional role as the filamentous hemagglutinin, a virulence effector molecule in B. pertussis. [file 1471-2164-9-566-S7.doc]
