# Supplementary material for: Genomic islands from five strains of Burkholderia pseudomallei
Source: BMC Genomics. 2008 Nov 27;9:566. doi: 10.1186/1471-2164-9-566 (PMC2612704; doi:10.1186/1471-2164-9-566)
Supplement: Additional file 8 — PCR primers.Table S5. PCR primers used in this study. [file 1471-2164-9-566-S8.doc]

Table S5. PCR primers used in this study

| Genomic islands | Genes or genomic locations | Primer IDs | Primer sequence 5'🡪.3' | Purpose |
| --- | --- | --- | --- | --- |
| GI14, GI14.1 | BPSS0654, BURPS1710b_A2222, BURPS1106A_A0882, BURPS668_A0974, BURPS305_5591 | bpss0654_forward | ACATCAAGGCCACATTGTCAGTGG | Presence/absence of a common metabolic gene |
|  |  | bpss0654_reverse | TGAAATCGTAGTCCGTGTTCGGCA |  |
| GI14a | BURPS305_5421 | burps305_5421_forward | GTGGCCGCAAGTTTCATCGAGTTT | Presence/absence of a specific metabolic gene |
|  |  | burps305_5421_reverse | GCCATGAAATCATCGTGCTTCGCT |  |
| GI5a, GI5a.1, GI5a.2 | *fhaB* cluster I: BURPS1710b_1277, BURPS1106A_1129, BURPS668_1122, BURPS305_2767 | fhaB_cluster 1_forward | TCGCTCAATACCGATGGGATGGT | Presence/absence of a potential virulence gene |
|  |  | fhaB_cluster 1_reverse | TATTGGACGTAATCGTCGCGTGGT |  |
| GI11.1 | *fhaB* cluster II: BURPS1106A_3880 | fhaB cluster 2_forward | GTCGACTATGCAAAGTCAGGCT | Presence/absence of a potential virulence gene |
|  |  | fhaB cluster 2_reverse | CGGCATCGTATAGCGATAGAG |  |
| GI16, GI16.1 | *fhaB* cluster III: BPSS2053, BURPS1710b_A1170, BURPS1106A_A2802 | fhaB cluster 3_forward | CATTATCGGCGGCAACCCGAATTT | Presence/absence of a potential virulence gene |
|  |  | fhaB cluster 3_reverse | CGAAATCAGGCCTTGGTTGACGTT | Presence/absence of a potential virulence gene |
| GI15d | *bpaA* (BURPS305_5906) | bpaA_forward | AACAATCAAGGCAATGATCCGGGC | Presence/absence of a potential virulence gene |
|  |  | bpaA_reverse | ATGTTCGGGACGATCACCACATCA |  |
